# Co-Encapsulation of Methylene Blue and PARP-Inhibitor into Poly(Lactic-Co-Glycolic Acid) Nanoparticles for Enhanced PDT of Cancer

**DOI:** 10.3390/nano11061514

**Published:** 2021-06-08

**Authors:** Jéssica A. Magalhães, Denise C. Arruda, Maurício S. Baptista, Dayane B. Tada

**Affiliations:** 1 Nanomaterials and Nanotoxicology Laboratory, Institute of Science and Technology, Federal University of São Paulo (UNIFESP), São Paulo 12231-280, Brazil; jessica.magalhaes@unifesp.br; 2 Laboratory of Experimental Cancer Biology, University of Mogi das Cruzes (UMC), São Paulo 08780-911, Brazil; denisearruda@umc.br; 3 Department of Biochemistry, Institute of Chemistry, University of São Paulo (USP), São Paulo 05508-000, Brazil; baptista@iq.usp.br

**Keywords:** PLGA nanoparticles, PARP inhibitor, photodynamic therapy

## Abstract

The development of resistance against photodamage triggered by photodynamic therapy (PDT) is ascribed mainly to the cellular redox defenses and repair. If the tumor tissue is not promptly eliminated by the first few PDT sessions, PDT-resistance can be favored, challenging the efficacy of the treatment. Although the mechanism of PDT resistance is still unclear, in vitro assays have evidenced that it can be developed through the PARP damage-repair signaling pathway. Therefore, inhibition of poly(adenosine diphosphate (ADP)-ribose) polymerase (PARP) has the potential to increase PDT efficacy. This work reports on the synthesis of a controlled release system of a photosensitizer, methylene blue (MB) and a PARP-inhibitor, the veliparib. MB and veliparib were co-encapsulated in poly(lactic-co-glycolic acid) (PLGA) nanoparticles (VMB-NPs). A colloidal stable aqueous suspension of nanoparticles was obtained. The average hydrodynamic diameter was 90 nm and a narrow size distribution was obtained, with a polydispersity index (PDI) of 0.08. The release kinetics of MB and veliparib from VMB-NPs showed an initial burst of 8.7% and 58.3% release of the total amounts of MB and veliparib respectively, in the first 6 h, and a delayed release of up to 11.3% and 70%, in 19 days, for MB and veliparib, respectively. The VMB-NPs showed no cytotoxicity in the dark but the viability of B16F10-Nex2 cells decreased by 36% when the cells were irradiated (102 J/cm^2^, 660 nm) and treated with VMB-NPs containing 1.0 µM of MB and 8.3 µM of veliparib. Considering the increased photoactivity even at low MB and veliparib concentrations and the absence of cytotoxicity in dark, the co-encapsulation of MB and veliparib was shown to be a promising strategy to improve the PDT efficacy.

## 1. Introduction

Chemotherapy is the therapeutic protocol most recommended for cancer treatment. However, despite causing damages to tumor cells, the biodistribution of the anticancer drugs compromises their effective concentration in tumor tissues. Thus, a high dose of these drugs is required, and they affect not only tumor cells, but also healthy cells, causing severe side effects. An alternative cancer treatment is photodynamic therapy (PDT), which is a minimally invasive procedure with less intense side effects and higher tumor selectivity. PDT involves the local activation of a photosensitizer (PS) by light irradiation in the presence of molecular oxygen. After PS activation, several photochemical pathways are triggered generating reactive oxygen species (ROS) which are responsible for inducing cell damage and cell death [1,2,3].

The efficiency of PDT depends on the combination of the intrinsic characteristics of PS, oxygen concentration and the PDT parameters, such as irradiation dose. The success of PDT treatment can be afforded by a PS that promotes low toxicity in the dark and high phototoxicity. However, the major portion of the common PSs aggregate in solution and consequently show limited biodistribution and rapid evasion from the tumor site. Besides that, most PSs may react with biomolecules and reach the tumor tissue in their inactive form. The combination of all these factors lead to the low effective concentration of PS in the tumor and low efficiency of PDT treatment.

The aforementioned limitation of most common PSs can be overcome by the encapsulation of the PS into nanoparticles (NPs). The NPs can protect the encapsulated PS from aggregation and deactivation and improve PS circulation time. In addition, small NPs easily accumulate in tumor tissues due to the unique anatomical and pathophysiological abnormalities of these tissues, including defective vascularity, extensive angiogenesis and an impaired lymphatic drainage system [4,5]. This phenomenon is known as the enhanced permeability and retention (EPR) effect and it is more pronounced when the NPs have a size in the range of 30–100 nm [6,7,8,9]. These delivery systems can improve the efficacy of the PDT-based cancer treatment and minimize the side effects when compared to traditional chemotherapy [10,11]. The application of NPs as a PS delivery system may also be a convenient strategy to promote subcellular targeting. The accumulation of PS in a specific cell compartment has been pointed as an important perspective to modulate the mechanism of cell death and to optimize PDT efficacy [12,13,14].

Different mechanisms of cell death can be triggered by PDT: apoptosis, necrosis, autophagy and paraptosis/mitotic catastrophe. It is noteworthy that more than one mechanism can be triggered at the same time, depending on the PS, treatment dose, type of cell and PS cytolocalization [12,13,15]. The use of a combined protocol involving two or more types of PS has shown to be a valuable way to enhance PDT efficacy [12,13,16]. The synergistic activity of PS was shown to be afforded by the simultaneous subcellular targeting of mitochondria, lysosomes and endoplasmic reticulum. Lysosomal damage has been associated with increased cell death through apoptosis due to the Ca^2+^ releasing into the cytoplasm, activation of calpain, and the consequent cleavage of ATG5 into pro-apoptotic fragments. Therefore, after lysosomal damage, cells become more sensible to photodamage which has been also attributed to the release of ferric ions and the triggered ROS production in the mitochondria. The optimization of PDT by a combined protocol can also be considered an approach to overcome the effects of hypoxia. Considering two PSs with different cytolocalization, the lysosomal photodamage may be induced right before mitochondrial damage and as a consequence, the pro-apoptotic agents have a longer lifespan and better performance [13,17,18]. Moreover, the combined use of two or more types of PS may also evoke simultaneously different pathways of cell death. Since some signaling agents released during PDT treatment can also trigger mechanisms of cell defense by inhibiting one pathway of cell death, tumor eradication may be assured by the photoactivation of an alternative mechanism of cell death [13,19].

Even if the NPs have improved PDT efficiency, their use has still not been able to avoid the development of the cell resistance to PDT, which is one of the main limiting factors to the complete elimination of the tumor tissue by PDT. The mechanisms of resistance to PDT have been associated to the general mechanisms of drug resistance as well as to specific properties of the PS. Malignant cells surviving PDT have proven to be more aggressive than the initial tumor population and therefore PDT protocols that lead to the more effective tumor eradication have been a subject of extensive research.

Numerous signaling pathways are triggered after PS photoactivation, which may promote or mediate cell death or even promote the repair and tolerance of damage [12,14,18,20]. The predominance of one mechanism of cell death over another depends on several factors, such as the molecular structure and concentration of the PS, its cytolocalization and also the irradiation dose. Some studies reported on the high apoptotic rates caused by the use of low irradiation doses [21,22], whereas high irradiation doses lead to necrosis [23]. According to Korbelik [23], this is due to the strong relationship between the cell damage caused by PDT and the activation and/or deactivation of poly(adenosine diphosphate (ADP)-ribose) polymerase (PARP). PARP is a group of proteins present in the nucleus of cells that participate in several mechanisms of cell maintenance, such as the processes of recovery and death. PARP plays a key role in the induction of apoptosis and autophagy. However, further investigation is needed in order to elucidate the relationship between cellular changes and PDT resistance [24]. The association between the overexpression of PARP-1, the extension of photodamage, and the development of cell resistance to PDT have been evidenced by Kim and co-workers [24]. In the case of limited DNA damage, PARP ribosylation facilitates DNA repair and contributes to cell survival and maintenance. In this sense, the control of apoptosis and autophagy pathways by PARP-1 can at the same time enhance PDT effects and also promote resistance to PDT [24]. Some researchers have reported on the combined application of PSs and PARP inhibitors, such as veliparib, and it was shown to be a valuable strategy to avoid cell recovery after PDT, improving the therapeutic effects [24,25,26]. Notoriously, the encouraging results observed in vitro could not be seen in vivo. Once in the blood circulation, each type of molecule (PS or PARP inhibitor) would show a specific circulation time and biodistribution and it would compromise the success of the treatment.

Therefore, in the present work, NPs containing co-encapsulated MB and PARP inhibitor were developed for the first time as a strategy to improve PDT efficiency by not only protecting the PS and improving its biodistribution but also by avoiding the development of PDT resistance through the simultaneous delivery of the PS and PARP inhibitor in situ. The use of co-encapsulated drugs may be an interesting way to improve the effects of PDT-based cancer treatment. The polymer poly(lactic-co-glycolic acid) (PLGA) was chosen to prepare the NPs due its biocompatibility and biodegradability. To the best of our knowledge, this is the first work that reports on the co-encapsulation of a PS and a PARP inhibitor in PLGA NPs. We expect that our findings may contribute to the development of an efficient system for treating melanoma, fueling new discussions about the therapeutic action of NPs with the co-encapsulation of active molecules towards the overcome of the resistance to PDT.

## 2. Materials and Methods

### 2.1. Materials

MB (Synth, Diadema, Brazil) was used as a photosensitizer molecule and veliparib (MedChem Express, Monmouth Junction, NJ, USA) was used as a PARP inhibitor. The polymers poly (vinyl alcohol) (PVA) and PLGA (50:50, Mw 7000–17,000) were supplied by Carbomer Inc (San Diego, CA, USA) and Sigma Aldrich (San Luis, MO, USA), respectively. Deionized water (Direct Q 3 UV, MilliQ, Merck Millipore, Burlington, MA, USA) was used in order to prepare all the samples. All the organic solvents were purchased from Synth (Diadema, Brazil). For the in vitro results, RPMI medium (Gibco, Thermo Fisher Scientific, Waltham, MA, USA) was prepared in deionized water. Fetal bovine serum (FBS) was used to supplement the medium and was purchased from Vitrocell Embriolife (Campinas, Brazil). Streptomycin, ampicillin, 3-(4,5-dimethylthiazol-2-yl)-2,5-diphenyltetrazolium bromide (MTT) and dimethylsulphoxide (DMSO) were supplied by Sigma-Aldrich (San Luis, MO, USA).

### 2.2. Synthesis of PLGA NPs

PLGA NPs without any encapsulated molecule were prepared by using a single emulsion-solvent evaporation method as described elsewhere [27]. Firstly, an organic solution of 100 mg of PLGA in 4 mL of DMF was prepared. In a second step, an aqueous solution was prepared by dissolving 200 mg of PVA in 20 mL of deionized water. The deionized water (type I) was further purified by using a 0.2 µm microfilter. This solution was kept under magnetic stirring and heated at 60 °C until complete solubilization of the PVA. After cooling at room temperature, the organic solution of PLGA was added dropwise to the aqueous solution under stirring. The solution was stirred overnight. Finally, the suspension was centrifuged at 1500× *g* and 4 °C for 20 min, and the supernatant was centrifuged at 15,000× *g* and 4 °C for 20 min. The resulting pellet was suspended in PBS solution (pH 7.4) and stored at −20 °C for further analysis.

### 2.3. Synthesis of PLGA NPs with MB and Veliparib Co-Loaded (VMB-NPs)

**Figure 1:**
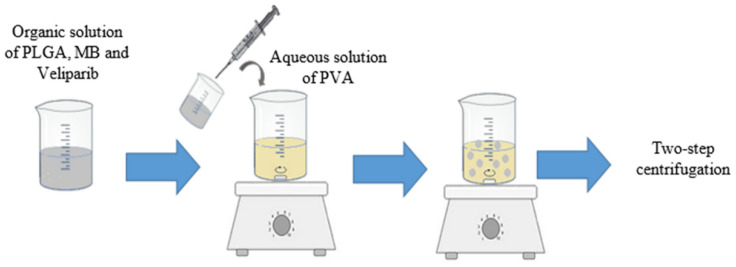
Synthesis of methylene blue and veliparib co-encapsulated into PLGA NPs (VMB-NPs) by using an adaptation of the nanoprecipitation method.

MB and veliparib were co-encapsulated in PLGA NPs by using an adaptation of the nanoprecipitation method, since it is the most indicated method to encapsulate hydrophobic drugs, such as veliparib [28,29]. Besides, this method was reported before as being a great approach to achieve a high encapsulation efficiency of MB into PLGA NPs [30]. VMB-NPs (Figure 1) were synthesized by an adaptation of the method reported by Jinwal and co-workers [30]. In brief, 3 mg of veliparib and 100 mg of PLGA were dissolved into 3 mL of acetone with an addition of 100 µL of a MB solution in ethanol (40 mg/mL). This solution was added dropwise to an aqueous solution of 1% (*w*/*w*) PVA. The solvent was evaporated at room temperature overnight under magnetic stirring. In order to washout the molecules that were not encapsulated the NPs suspension was centrifuged at 1500× *g* and 4 °C for 20 min. Then, the supernatant was centrifuged at 15,000× *g* and 4 °C for 20 min. The resulting pellet was suspended in PBS solution (pH 7.4) by sonication and stored at −20 °C for further analysis.

### 2.4. Evaluation of NPs Physicochemical Properties

The particle size and zeta potential of the NPs were measured by dynamic light scattering technique (DLS, DelsaNano C, Beckman Coulter, Brea, CA, USA). The samples were diluted in type I deionized water. The molecules concentration into the NPs were determined by UV-Vis spectroscopy (Jasco V-730, Indaiatuba, Brazil). Raman spectrum analysis was performed by using a high-sensitivity laser microscope confocal Raman spectrometer (LabRam HR Evolution, Horiba Scientific, Jundiaí, Brazil). Raman peak analysis was done by plotting the spectra using Origin software and comparing the number of lateral peak shifts of NPs aqueous suspensions (PLGA NPs and VMB-NPs) and free molecules powder spectra.

### 2.5. Determination of Encapsulation Efficiency (%EE)

The drugs encapsulation efficiency in PLGA NPs was determined by UV-Vis. The concentration of the drugs was calculated by using a calibration curve of concentration vs absorbance. The calibration curves were previously determined by using several solutions of different concentration of each molecule in methanol. NPs were collected by centrifugation (15,000× *g*, 4 °C for 20 min) and the pellet was solubilized in methanol. The absorbance of the resultant solution was measured at 650 and 290 nm for MB and veliparib respectively. The encapsulation efficiency was obtained by using the following equation:

%EE = (W_NPs_/W_s_) × 100%, (1)

where W_NPs_ represents the mass of the drug loaded in PLGA NPs and W_s_ the mass of the molecule added during the NPs synthesis.

### 2.6. In Vitro Drug Release

The kinetics of release of MB and veliparib from PLGA NPs was monitored in PBS solution. Firstly, samples of 2 mL of NPs suspension were centrifuged (21,380× *g*, 4 °C for 20 min). Then, NPs were resuspended with fresh PBS and incubated in a 5% CO_2_ atmosphere at 37 °C. At different time intervals, samples were centrifuged and the supernatant was collected. The NPs were resuspended in PBS and the absorbance of the collected supernatant was measured by UV-Vis spectroscopy (λ_MB_ = 665 nm and λ_veliparib_ = 270 nm). Concentration of MB and veliparib was calculated by using the molar extinction coefficient of MB (95,000 M^−1^ cm^−1^) [31] and veliparib (5087 M^−1^ cm^−1^).

### 2.7. Cell Viability Assays

Murine melanoma cell line B16F10-Nex2 was a gift from Prof. Dr. Luiz R. Travassos from Federal University of São Paulo, Brazil. The cells were cultured in RPMI medium supplemented with 10% (*v*/*v*) FBS, streptomycin (0.1 g/L), and ampicillin (0.025 g/L). Cells were incubated at 37 °C in a humidified atmosphere with 5% CO_2_. After a period, cells were seeded into 96-well plates (4 × 10^3^ cells per-well) and incubated for 24 h. Afterwards, RPMI medium was removed and replaced by a fresh one, containing different samples. At first, cell viability was evaluated after cell incubation with free molecules at various concentrations. Cells were treated individually with MB and veliparib in its free form (0.1 µM and 2.0 µM, respectively), as positive controls, and also incubated with a combination of MB (0.1 µM) and different concentrations of veliparib (0.5–2.0 µM). MTT assay was also performed after encapsulation of both active molecules into PLGA NPs. In this case, cells were incubated with free MB (9.3 µM) and with VMB-NPs at different concentrations (1.5, 3.7 and 9.3 µM of MB). For both experiments, the incubation time with the samples was about 12 h. After this period, 100 µL of PBS were added in each well and cells were irradiated (102 J/cm^2^) by using an LED-coupled irradiation chamber (IrradLed, Biopdi, São Carlos, SP, BR), with maximum emission at 660 nm. The irradiated and non-irradiated cells were further incubated in culture medium for 12 h. The dark and phototoxic effects of the samples on cells were evaluated after incubation with 100 µL of a MTT solution (0.5 mg/mL) for 3 h. The final absorbance of formazan was measured after solubilizing formazan crystals in DMSO. The measurement was carried out in a microplate reader (BioTek, Winooski, VT, USA) at 540 nm. The absorbance of cells in absence of irradiation was considered as 100% of viability.

## 3. Results and Discussion

### 3.1. Physicochemical Properties

Considering the previously reported in vivo lower efficiency despite of the encouraging results obtained in vitro of the combined use of PS and PARP inhibitors, our research was focused on the co-encapsulation of a PS and a PARP inhibitor into a NP. Polymeric NPs were used in order to guarantee the simultaneous delivery of both PS and PARP inhibitor inside the tumor. The polymer poly(lactic-co-glycolic acid) (PLGA) was chosen to prepare the NPs due its biocompatibility and biodegradability in addition to a flexibility for tailoring chemical composition and different morphologies [32,33].

**Figure 2:**
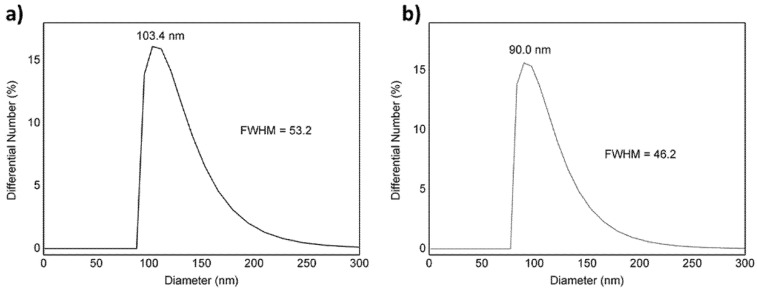
Size distribution of (**a**) poly(lactic-co-glycolic acid) (PLGA) NPs and (**b**) VMB-NPs.

In order to evaluate the effect of the MB and veliparib loading on NPs parameters, the physical characteristics of the NPs, such as hydrodynamic diameter, polydispersity index (PDI) and zeta potential were measured in the presence and absence of the active molecules (Table 1). As shown in Figure 2, PLGA NPs suspension presented low polydispersity, which is indicated by the narrow size distribution and by the low value of full width at half maximum high (FWHM). The encapsulation of MB and veliparib resulted in smaller NPs in comparison with PLGA NPs without any encapsulated molecule. The average hydrodynamic diameter of VMB-NPs was 90 nm, which was 13% smaller than PLGA NPs. A narrow size distribution curve and low value of FWHM value also indicated low polydispersity (PDI = 0.08).

Despite the high colloidal stability, zeta potential for both NPs was low. However, loading MB and veliparib into PLGA NPs increased the zeta potential in 46% compared with PLGA NPs. As already known, several synthesis parameters of PLGA NPs, as polymer concentration and organic solvent, affect particle size and surface properties [34]. In our system, the decrease in size was probably promoted by MB/veliparib-polymer interactions, which could compact the polymeric matrix [30]. This change in size by loading PLGA with MB and veliparib was a positive result, since the EPR effect is promoted by NPs in the range of 30–100 nm [6,7,8,9]. Additionally, the low value of zeta potential could improve the NPs performance as controlled delivery systems. There is evidence that NPs with zeta potential values close to the neutral charge reduce unexpected interactions with proteins and blood vessels, promoting better effectiveness in drug biodistribution [35,36].

**Figure 3:**
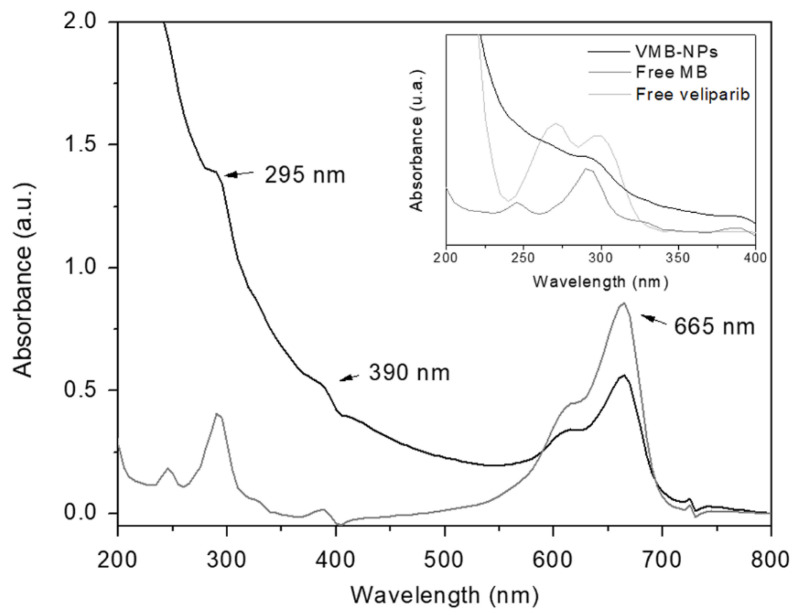
UV-Vis spectra of VMB-NPs (black) and free methylene blue (MB) (dark gray). Insert: UV-Vis spectra of VMB-NPs (black), free MB (dark gray) and free veliparib (gray) at smaller wavelengths.

The encapsulation of MB and veliparib into PLGA NPs was confirmed by UV-Vis spectroscopy. The presence of both molecules in PLGA NPs was evidenced by comparing the absorbance spectra of free veliparib, free MB and VMB-NPs (Figure 3). The MB incorporation in PLGA NPs was indicated by the characteristic band of MB monomers at 665 nm. An absorption band was also noticed in the VMB-NPs spectrum at smaller wavelengths (Insert Figure 3). The absorption band at 295 nm suggests the overlap of the MB and veliparib absorption bands (λ_veliparib_ = 300 nm and λ_MB_ = 290 nm). At smaller wavelengths, veliparib also shows another absorption band at 270 nm that could not be observed in the VMB-NPs spectrum due to the intense light scattering at wavelengths lower than 300 nm.

UV-Vis spectra were also used to calculate the encapsulation efficiency of both molecules. The encapsulation efficiency (%EE) was 23% and 58% for MB and veliparib, respectively. Despite the low encapsulation of MB, the %EE value obtained here was very close to that one obtained by Jinwal and co-workers [30], by using a similar protocol. It is worth mentioning that considering the moderate hydrophilic nature of MB, an attempt of adding MB in the aqueous phase during the synthesis of PLGA NPs was also performed aiming at higher values of %EE. Nevertheless, its encapsulation into PLGA NPs has not been confirmed by UV-Vis spectroscopy, since no absorption bands were observed. The higher %EE of veliparib in comparison with MB can be attributed to the higher hydrophobicity of the molecule which favors the incorporation by the nanoprecipitation method [28,29].

**Figure 4:**
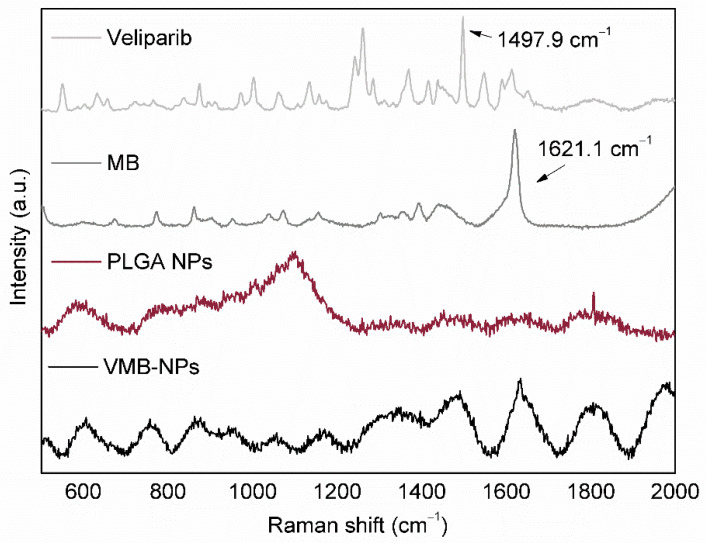
RAMAN spectra of NPs suspensions (PLGA NPs and VMB-NPs), and MB and veliparib powders.

VMB-NPs were further characterized by RAMAN spectroscopy. The RAMAN spectra (Figure 4) showed all the main peaks observed in the spectrum of each component individually (1621.1 cm^−1^ correspondent to MB and 1497.9 cm^−1^ correspondent to veliparib). This result evidenced that inside the NPs there was no molecular interaction between MB and veliparib. The absence of the mentioned intermolecular interactions was considered a positive feature of the NPs since any molecular interaction would affect the molecules release from the NPs by changing the diffusion rate of the molecules or the kinetics of polymer biodegradation by hydrolysis.

### 3.2. In Vitro Drug-Release

**Figure 5:**
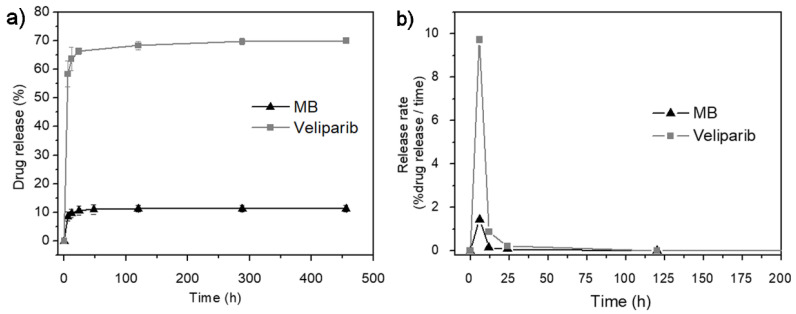
(**a**) Drug release profile of MB and veliparib from VMB-NPs. (**b**) Release rate of MB and veliparib from VMB-NPs as a function of time.

The kinetics of molecules release from the matrix is of crucial importance to the therapeutic efficiency of the system. The VMB-NPs presented herein may provide simultaneous photoactivation of MB and the PARP inhibition by veliparib. Therefore, the drug release profile of each drug was measured in vitro. The release profile of MB and veliparib is presented in Figure 5a. Only 11.3% of the total MB content was released during all the analysis time, suggesting a slow kinetic release. Similar behavior was observed for veliparib, but in this case, the initial burst release was more pronounced. The burst release effect was observed for both molecules after the first 6 h of incubation. After this time, 8.7% of MB was released while the release of veliparib was of 58.3%. The slower release behavior of MB was reported to be a result of its poor encapsulation by the nanoprecipitation method. In this way, some authors [30,37,38,39] have suggested different strategies to promote a higher encapsulation of MB and then a faster release in aqueous solution. Klepac-Ceraj and co-workers have noticed that changes in NPs surface charge would be a valuable strategy to modulate the release kinetics of MB. The incorporation of MB into cationic NPs resulted in a more accentuated burst release effect and 80% of MB was released after 12 h. Meanwhile, MB incorporation into anionic NPs exhibited a slower release kinetic and a release of only 28% was reached after the first 12 h of incubation [37]. The encapsulation of veliparib has been addressed only by few researchers. Muñoz-Gámez and co-workers reported on the association of veliparib with a magnetic NPs of Fe_3_O_4_/Fe core and a final layer of SiO_2_. The release profile did not present a well-defined burst release effect and the release of veliparib was gradual. A release of 50% was observed after 15 h of incubation of the NPs in aqueous solution [40].

Besides the surface modifications, other factors such as type of molecule, molecules concentration inside the NPs, drug-polymer and drug-drug interactions, and the matrix composition have been reported to promote more uniform dispersion of the drug into the matrix and consequently modify release kinetics [41]. Moreover, the release kinetic is also dependent on the mechanism of matrix degradation [30,40]. Herein, various processes may contribute to the release of the entrapped MB and veliparib from VMB-NPs. Since PLGA is a biodegradable polymer, the kinetic release not only depends on the diffusion rate of the molecule, but also on the rate of water penetration into the PLGA matrix as well as the erosion and diffusion of PLGA fragments. The erosion of these small polymeric fragments alters the pH gradient formed by the PLGA hydrolysis, accelerating the diffusion rate of the entrapped molecule [41].

Notably, the rate of release of MB and veliparib from VMB-NPs showed the same profile in the function of release time (Figure 5b). It was evidenced that the maximum release rate was obtained at the same time (6 h) for both molecules which is a suitable property to the dual release system proposed herein, since it is an indicative that VMB-NPs could provide maximum concentration of MB and veliparib ensuring simultaneous PDT and PARP inhibition therapy. Furthermore, after the maximum release, the release profile assumes a zero-order kinetics which is important to provide the continuous and simultaneous release of MB and veliparib. Consequently, PDT and the PARP inhibition could be simultaneously activated until the total biodegradation of PLGA and/or release of molecules.

The release mechanism of encapsulated drugs from the PLGA matrix may be very complex and difficult to understand. Generally, when in aqueous solution, the PLGA matrix absorbs water that penetrates from the surface to the center of the NPs. This matrix hydration may activate the PLGA hydrolysis and then it promotes the release of the encapsulated drugs. Nonetheless, other processes may contribute to the release mechanism, such as degradation rate, erosion and diffusion of PLGA fragments and diffusion rate of the drugs [41]. Furthermore, release behavior also depends on the drug solubility; while the release of hydrophilic drugs manly occurs by diffusion, the release of hydrophobic drugs is normally associated with swelling and matrix erosion [42]. So, the co-encapsulation of a hydrophilic and hydrophobic molecule into the same matrix brings an additional complexity to the system since although the molecules do not interact with each other directly, each one can induce changes in the external medium. As a consequence, the diffusion and solubility of the encapsulated molecules as well as the erosion/degradation of the polymeric matrix are different in the modified medium. Therefore, there is still no appropriate mathematical model for the delivery system reported herein and it could be an interesting subject of research for researchers from the field of mathematical modeling.

### 3.3. Evaluation of Cell Response to Non-Encapsulated Molecules and VMB-NPs

**Figure 6:**
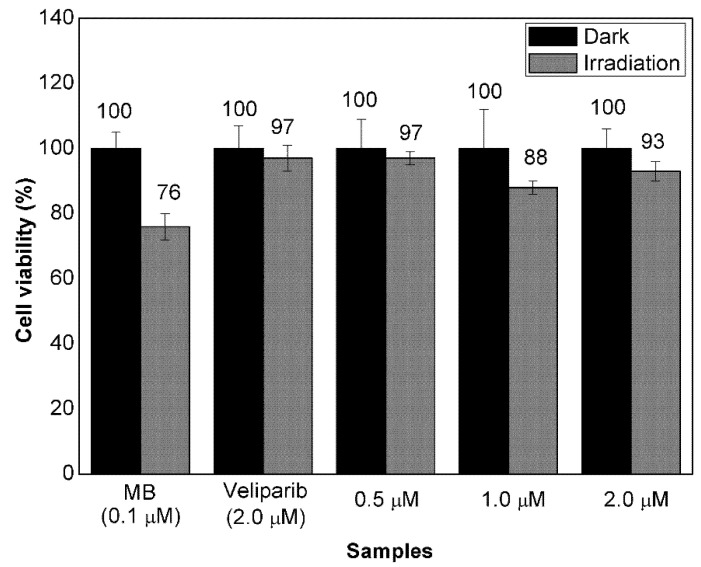
Viability of B16F10-Nex2 cells after incubation with free MB and free veliparib. Both molecules were incubated individually at 0.1 and 2.0 µM, respectively. The cells were also treated with a solution containing MB and veliparib at different concentrations ((MB) = 0.1 µM and (veliparib) = 0.5, 1.0 and 2.0 µM). Cell were kept in the dark or irradiated (102 J/cm^2^, 660 nm). Values are depicted as mean values and standard deviation (*n* = 3).

The use of a PARP inhibitor may lead to changes in cell responses usually observed in therapeutic protocols based on PDT. For this reason, the photoactivity of MB was evaluated by measuring the viability of B16F10-Nex2 cells after incubation with MB (0.1 µM) combined with veliparib at different concentrations. For comparative purposes, the viability of cells incubated with MB and veliparib separately was also measured. As shown in Figure 6, the viability of cells incubated with veliparib only or combined with MB after irradiation was in the range of 88–97%. The lowest average viability (88%) was observed with the cells incubated simultaneously with MB and veliparib at 1.0 µM. Therefore, the use of MB (0.1 µM) and veliparib (1.0 µM) promoted a decrease of 9% in cell viability after laser irradiation when compared to the incubation with free veliparib (2.0 µM). Nevertheless, in comparison with the incubation with MB alone, the simultaneous incubation of veliparib and MB did not show enhanced photoactivity. On the contrary, the cell viability after irradiation of cells simultaneously incubated with MB and veliparib was higher than the viability of cells incubated with MB only (76%). This observation suggested that free veliparib has some sort of inhibition to photodamage caused by MB photoactivation. There may be innumerous reasons that can explain this effect, including different rates of cell uptake and chemical processes unrelated to the PARP activity, such as singlet oxygen suppression of indole derivatives [43].

Since the values of cell viability were very similar when comparing cells incubated with MB alone and with a combination of MB (0.1 µM) and veliparib at 0.5, 1.0, and 2.0 µM it could be reasonable to conclude that the use of MB in the presence of veliparib would not lead to an enhanced PDT treatment. However, despite the limited information regarding the combination of these two compounds in PDT, the literature in this field has pointed to innumerous factors associated with the mechanism of action of a PS and a PARP inhibitor when they are combined in the same PDT treatment. These factors include incubation time, dose of irradiation, relative concentration of PS and PARP inhibitor and even the addition order of each molecule. Herein, despite the simultaneous cell incubation of MB and veliparib, it would be plausible to expect both molecules to be cell internalized with different rates. In fact, the pre-incubation of cells with MB, followed by incubation with veliparib was also tested but it led to cell viability values even higher (see Supplementary Material—Figure S1, Table S1). These results outlined the importance of using a drug-carrier system such as VMB-NPs as a tool to assure simultaneous delivery of both PS and PARP inhibitor inside the cells.

Although this work addressed the use of MB at very low concentrations and veliparib at high concentrations, even at a concentration of veliparib about 385 times higher than the inhibitory constants of PARP-1 and PARP-2 [44], there are still several approaches to be explored that would lead to the enhanced efficiency of PDT, including the application of more than one time of PARP inhibitor. Despite the similarities in the catalytic activity of some PARP inhibitors, there are differences in their ability to trap PARP, as discussed by Stewart and co-workers [45]. Thus, herein, it has also to be considered that the low effect of veliparib on the MB photoactivity could be afforded by the extended time of veliparib in trapping PARP onto DNA which may have a strong relationship with the phototoxicity of the system during PDT.

**Figure 7:**
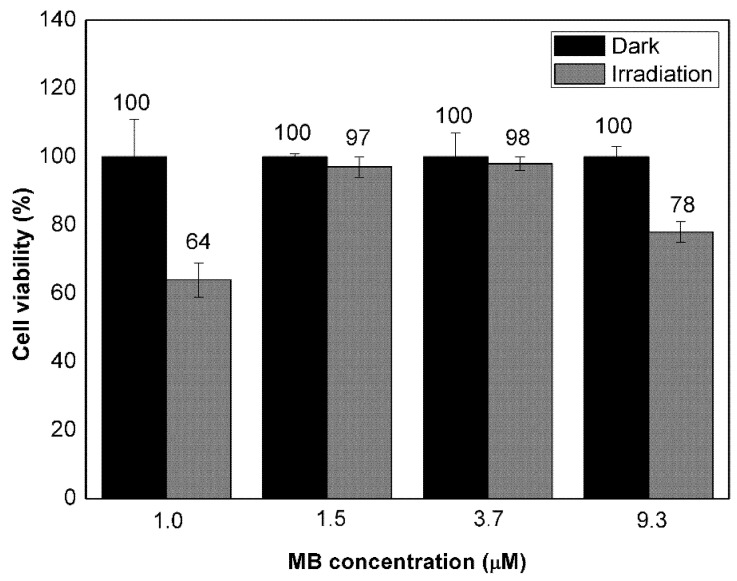
Viability of B16F10-Nex2 cells incubated with VMB-NPs containing encapsulated veliparib (8.3, 11.0, 28.0 and 69.0 µM) and MB (1.0, 1.5, 3.7 and 9.3 µM) at different concentrations in the dark and under laser irradiation (102 J/cm^2^, 660 nm). Values are depicted as mean values and standard deviation (*n* = 3).

The in vitro effect of VMB-NPs was also investigated but, in this case, both concentration of MB and veliparib were varied whereas the molar ratio of veliparib to MB was kept as 7.4 (Figure 7). The viability of B16F10-Nex2 cells incubated with VMB-NPs containing MB at 1.5 and 3.7 µM and veliparib at 11.0 and 28.0 µM respectively did not decrease under irradiation (102 J/cm^2^, 660 nm). Nevertheless, cells incubated with VMB-NPs containing MB and veliparib at 9.3 and 69.0 µM respectively, showed cell viability 22% lower after irradiation. In addition, when the molar ratio of veliparib to MB was slightly increased to 8.3 but the MB and veliparib concentrations were much lower (1.0 µM of MB and 8.3 µM of veliparib), photoactivity was further enhanced, resulting in a decrease of 36% of cell viability. Therefore, it was observed that not only the molar ratio but also the absolute concentration of MB and veliparib could change VMB-NPs photoactivity and, the combination of MB and veliparib at these concentrations into PLGA NPs could be considered as a strategy to enhance photodamage of B16F10-Nex2 cells by MB.

Regardless of the therapeutic effects of PARP inhibitors in cancer treatment, the relationship between PARP and the mechanisms of ROS generation by a PS during PDT has not been fully elucidated [46]. After PS photoactivation, several signaling pathways are triggered and may be related to mechanisms of cell death as well as cell survival [20]. This is also observed after PARP activation/deactivation. The role of PARP inhibitors in cell mechanisms depends on various factors, such as the extension of cell damage, concentration and irradiation dose applied during PDT. Furthermore, PARP inhibitors may also damage DNA by generating complexes even more toxic than the single-strand breaks caused by PDT [44]. Some mechanisms of cell death have even show to be PARP-dependent and the presence of PARP inhibitor increased cell viability after PDT treatment [12].

Clearly, further investigation of the simultaneous use of MB and veliparib in PDT is required in order to achieve enhanced PDT efficiency. Nevertheless, the VMB-NPs presented herein would be a clever strategy to achieve the suitable ratio of released MB and veliparib in the function of time and meanwhile promoting the enhanced tumor targeting due to the well-known EPR effect of NPs in cancer therapy.

## 4. Conclusions

This work reports on a dual-drug release platform (VMB-NPs) prepared by the co-encapsulation of MB and veliparib in PLGA NPs. The co-encapsulation did not affect the NPs size and stability. A stable colloidal suspension of 90 nm NPs was obtained. Although both veliparib and MB showed burst release after 6 h, the release kinetics suggested a controlled release during the next 450 h. The slow release at the time interval of 12 h provided the release of only 10% of MB and 64% of veliparib. The fast release of MB and veliparib at the initial steps may be a positive feature in the simultaneous treatment by PDT and PARP inhibition therapy. The MB released at the initial step may provide the photodamage of the cells while the PARP inhibitor released simultaneously and during the later stages will be important to inhibit the recovery of the photodamaged cells, enhancing the efficiency of the therapy. The in vitro assays with VMB-NPs indicated enhanced photoactivity when the encapsulation was performed with the combination of MB and veliparib at 1.0 and 8.3 µM, respectively. At this condition, the cell viability decreased 36% after photoactivation of VMB-NPs. Notoriously, current limited information on the mechanisms of cell death that are activated from the association of PS and PARP inhibitors in PDT has hampered the observation of greater therapeutic effect. However, this work addresses for the first time the application of a combined use of MB and PARP inhibitor by co-encapsulating these molecules in PLGA NPs. The results presented herein will be useful for driving future research towards the establishment of the best conditions of PDT treatment (light dose, PS concentration, incubation time and release kinetics) in order to reach higher efficiency of PDT at lower PS dose. Furthermore, by combining PARP inhibition with PS photodamage this work brings a new perspective to optimize PDT treatment.

## Figures and Tables

**Table 1:** Hydrodynamic diameter and zeta potential of PLGA NPs and VMB-NPs, and encapsulation efficiency of MB and veliparib.

Samples	Hydrodynamic Diameter (nm)	PDI	Zeta Potential (mV)	Encapsulation Efficiency (%)	
PLGA NPs	103.4	0.07 ± 0.03	−6.8 ± 0.6		
VMB-NPs	90.0	0.08 ± 0.03	−3.7 ± 0.2	MB 23	Veliparib 58

## Data Availability

Raw data were generated at ICT-UNIFESP. Derived data supporting the findings of this study are available from the corresponding author D.B.T on request.

## Supplementary Materials

The following are available online at https://www.mdpi.com/article/10.3390/nano11061514/s1, Figure S1: Viability of B16F10-Nex2 cells after incubation with free MB (10 μM) and free veliparib (20 μM) individually or combined. When the cells were treated with a combination of MB and veliparib, the incubation of cells with MB at 10 μM was performed 4h before the incubation with veliparib at 5, 10 and 20 μM. Cells were irradiated (102 J/cm^2^, 660 nm) and the values of cell viability are depicted as mean values and standard deviation (*n* = 3), Table S1: Values of concentration of free MB in solution and encapsulated into VMB-NPs required to induce about 50% of cell death in comparison with untreated cells in the absence of irradiation. Cells incubated with MB or VMB-NPs were irradiated (102 J/cm^2^, 660 nm).
