# Supplementary material for: Co-Encapsulation of Methylene Blue and PARP-Inhibitor into Poly(Lactic-Co-Glycolic Acid) Nanoparticles for Enhanced PDT of Cancer
Source: Nanomaterials (Basel). 2021 Jun 8;11(6):1514. doi: 10.3390/nano11061514 (PMC8227603; doi:10.3390/nano11061514)
Supplement: Supplementary file 1 [file nanomaterials-11-01514-s001.zip › nanomaterials-1139628-supplementary.pdf]

nanomaterials

MDPI

Supplementary Material

Co-Encapsulation of Methylene Blue and PARP-Inhibitor into Poly(Lactic-Co-Glycolic Acid) Nanoparticles for Enhanced PDT of Cancer

Jéssica A. Magalhães [1], Denise C. Arruda [2], Maurício S. Baptista [3] and Dayane B. Tada [1,*]

[1] Nanomaterials and Nanotoxicology Laboratory, Institute of Science and Technology, Federal University of São Paulo (UNIFESP), São Paulo 12231-280, Brazil; jessica.magalhaes@unifesp.br
[2] Laboratory of Experimental Cancer Biology, University of Mogi das Cruzes (UMC), São Paulo 08780-911, Brazil; denisearruda@umc.br
[3] Department of Biochemistry, Institute of Chemistry, University of São Paulo (USP), São Paulo 05508-000, Brazil; baptista@iq.usp.br
* Correspondence: d.tada@unifesp.br

This work addresses the application of PDT and PARP inhibition therapies to avoid the development of PDT-resistance by cancer cells. Nevertheless, the mechanisms of cell death/cell survival in this type of therapy have not been fully elucidated yet. The literature has shown that the role of PARP inhibitors in cell mechanisms depends on various factors, such as the extension of cell damage, concentration, and irradiation dose applied during PDT. Herein, in addition to the simultaneous incubation of B16F10-Nex2 melanoma cells with veliparib and MB, the pre-incubation with MB followed by incubation with veliparib after 4h was also tested. As can be seen in Figure S1, this treatment resulted in values of cell viability higher than the viability of irradiated cells in the absence of MB or veliparib. This result outlined that the addition order of PS and PARP inhibitor is also a factor that has to be settled to achieved better therapeutic efficiency. The works reported before had pointed out that PARP inhibitors may also damage DNA by generating complexes even more toxic than the single-strand breaks caused by PDT. Some mechanisms of cell-death have even shown to be PARP-dependent and the presence of PARP inhibitor increased cell viability after PDT treatment.

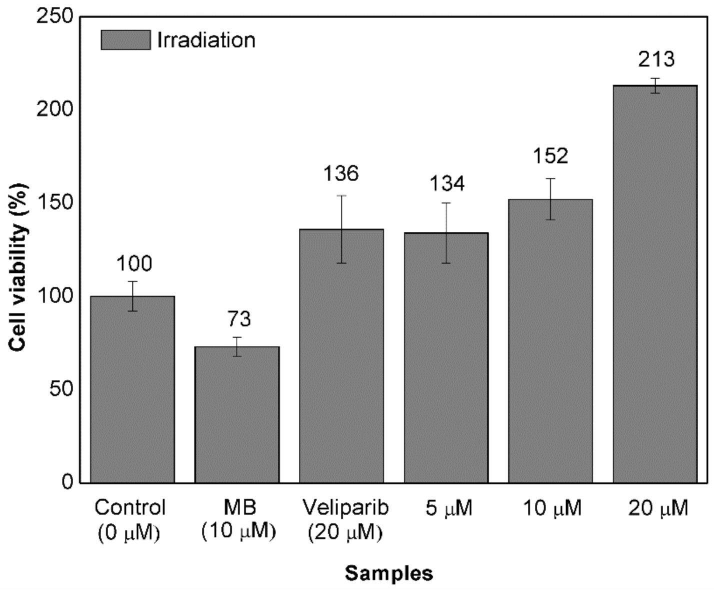

Figure S1. Viability of B16F10-Nex2 cells after incubation with free MB (10 μM) and free veliparib (20 μM) individually or combined. When the cells were treated with a combination of MB and veliparib, the incubation of cells with MB at 10 μM was performed 4h before the incubation with veliparib at 5, 10 and 20 μM. Cells were irradiated (102 J/cm^2, 660 nm) and the values of cell viability are depicted as mean values and standard deviation (n = 3).

Although the direct comparison between Figures 6 and 7 of the manuscript was hampered by the different experimental conditions applied in each assay, a simple analysis would indicate that there was no advantage of using VMB-NPs to enhance therapeutical efficiency. Nevertheless, the discussion has to be deeper by evaluating the adversities found with the use of free MB in vivo. As extensively pointed in the introduction section, it has been shown that free MB can be reduced by biomolecules and reach the tumor at its inactive form. Also, the inefficient MB cytotoxicity in vivo has to promote the development of PDT-resistant cells. Notoriously, it is important to have in mind that one of the most important advantages of a nanoparticulated system is the enhanced biodistribution and tumor target, which could not be evaluated without in vivo assay. Also, even if in vitro assays had not evidenced an advantage of using VMB-NPs over the application of free MB, in vivo assays may still show high therapeutical efficiency as it has already been seen in one of our previous work [1] with a similar drug-release system. Even so, in order to better compare free MB in solution and VMB-NPs photoactivity, it was added Table S1 to the supplementary material of the revised manuscript, pointing the minimum concentration of free MB and VMB-NPs to reach about 50% of cell viability. In this table, it is possible to see that in comparison with cells incubated in the absence of samples and the absence of irradiation, the concentration of MB to induce 51 ± 2% of cell death was 9.3 μM when free in solution and of 1.0 μM when encapsulated into VMB-NPs.

Table S1. Values of concentration of free MB in solution and encapsulated into VMB-NPs required to induce about 50% of cell death in comparison with untreated cells in the absence of irradiation. Cells incubated with MB or VMB-NPs were irradiated (102 J/cm^2, 660 nm).

Sample	MB Concentration (μM)	Cell Viability (%)
Free MB	9.3	51 ± 2
VMB-NPs	1.0	51 ± 4

1. Arruda D.C.; de Oliveira T.D.; Cursino P.H.F.; Maia V.S.C.; Berzaghi R.; Travassos L.R.; Tada D.B. Inhibition of melanoma metastasis by dual-peptide PLGA NPs. *Biopolymers* **2017**, *108*, e23029. https://doi.org/10.1002/bip.23029.
